# A therapeutic oxygen carrier isolated from *Arenicola marina* decreased *P. gingivalis* induced inflammation and tissue destruction

**DOI:** 10.1038/s41598-020-71593-8

**Published:** 2020-09-08

**Authors:** Fareeha Batool, Céline Stutz, Catherine Petit, Nadia Benkirane-Jessel, Eric Delpy, Franck Zal, Elisabeth Leize-Zal, Olivier Huck

**Affiliations:** 1 INSERM (French National Institute of Health and Medical Research), UMR 1260, Regenerative Nanomedicine, Fédération de Médecine Translationnelle de Strasbourg (FMTS), Strasbourg, France; 2 Faculté de Chirurgie-Dentaire, Université de Strasbourg, 8 rue Sainte-Elisabeth, 67000 Strasbourg, France; 3 Pôle de Médecine et Chirurgie Bucco-Dentaire, Hôpitaux Universitaires de Strasbourg, 67000 Strasbourg, France; 4 Hémarina SA, Aéropôle centre, 29600 Morlaix, France

**Keywords:** Dental diseases, Infection, Inflammation

## Abstract

The control of inflammation and infection is crucial for periodontal wound healing and regeneration. M101, an oxygen carrier derived from *Arenicola marina,* was tested for its anti-inflammatory and anti-infectious potential based on its anti-oxidative and tissue oxygenation properties. In vitro, no cytotoxicity was observed in oral epithelial cells (EC) treated with M101. M101 (1 g/L) reduced significantly the gene expression of pro-inflammatory markers such as TNF-α, NF-κΒ and RANKL in *P. gingivalis*-LPS stimulated and *P. gingivalis*-infected EC. The proteome array revealed significant down-regulation of pro-inflammatory cytokines (IL-1β and IL-8) and chemokine ligands (RANTES and IP-10), and upregulation of pro-healing mediators (PDGF-BB, TGF-β1, IL-10, IL-2, IL-4, IL-11 and IL-15) and, extracellular and immune modulators (TIMP-2, M-CSF and ICAM-1). M101 significantly increased the gene expression of Resolvin-E1 receptor. Furthermore, M101 treatment reduced *P. gingivalis* biofilm growth over glass surface, observed with live/dead analysis and by decreased *P. gingivalis* 16 s rRNA expression (51.7%) (*p* < *0.05)*. In mice*,* M101 reduced the clinical abscess size (50.2%) in *P. gingivalis*-induced calvarial lesion concomitant with a decreased inflammatory score evaluated through histomorphometric analysis, thus, improving soft tissue and bone healing response. Therefore, M101 may be a novel therapeutic agent that could be beneficial in the management of *P. gingivalis* associated diseases.

## Introduction

Periodontitis is an inflammatory disease of infectious origin characterized by destruction of tooth supporting tissues including alveolar bone, gingiva, periodontal ligament and cementum^1^. Severe periodontitis has been ranked as the 6th most prevalent disease worldwide affecting around 743 million people^2^. Moreover, it has been associated with reduced quality of life and is considered as one of the main causes of tooth loss^3^. Clinically, such disease manifests itself as inflamed and bleeding gums, periodontal pocket formation, periodontal tissue and alveolar bone destruction, leading to tooth mobility and eventual tooth loss^4^.

Initiation of periodontitis is associated with the development of oral biofilm over teeth and gums resulting in low-grade inflammation with no evident clinical signs. Failure to disrupt this biofilm actuates a change in oral microenvironment, thereby shifting the balance from symbiotic to a predominantly dysbiotic microbial flora, consequently inducing an exacerbated inflammatory response^5,6^. In this regard, *Porphyromonas gingivalis* (*P. gingivalis*), a gram negative anaerobe, is considered as one of the keystone pathogens^7,8^. It acts through its various virulence factors such as lipopolysaccharide (LPS), fimbriae and gingipains to hijack host immune response leading to sustained inflammation and subsequent tissue destruction, at local as well as distant sites^9^.

A large variety of cell types are present in the periodontal wound environment such as epithelial cells (EC), fibroblasts (FB), osteoblasts (OB) and immune cells^10,11^. In case of foreign aggression, EC forming the junctional epithelium, act as a physical barrier and are the first line of host defense owing to their ability to elicit an innate immune response^12^. *P. gingivalis* invasion of EC activates resident periodontal tissue cells which in turn affects cell proliferation, differentiation, and migration of precursor immune cells into the wound environment^13–15^. This induces production of various inflammatory and immune mediators, such as cytokines, chemokines and matrix metalloproteinases by periodontal cells, resulting in tissue destruction^16^. The key pro-inflammatory markers, tumor necrosis factor alpha (TNF-α) and interleukin-1 beta (IL-1β), play a prominent role in orchestrating inflammation by the induction of several other inflammatory mediators and chemokines through nuclear factor kappa-B (NF-κΒ) associated pathways^16–18^. The intricate balance between the pro-inflammatory molecules counteracted by anti-inflammatory and pro-resolution mediators, such as platelet-derived growth factor (PDGF), transforming growth factor-beta (TGF-β), IL-10, and resolvins, determines the outcome of wound healing process^19–21^. Therefore, control of inflammation and infection are crucial to the success of periodontal treatment^20^.

Conventionally, periodontitis is treated, depending on the disease severity, through non-surgical or surgical treatment approaches^22^. The former approach includes mechanical debridement to achieve root surface decontamination and is associated with a high rate of success characterized by reduction in the number of diseased sites, tissue inflammation and pocket depth^23^. However, in severe cases with deep and infrabony defects, either periodontal surgery and/or local or systemic pharmacotherapy is warranted^22^. In this context, several natural and synthetic compounds, and novel combinations of drugs with local drug delivery scaffolds have been developed and tested to improve treatment outcomes with tissue-targeted drug delivery^24–29^.

HEMARINA-M101 (M101) is a natural extracellular hemoglobin isolated from the marine lugworm *Arenicola marina*. This extracellular oxygen carrier is a biopolymer of high molecular weight (~ 3,600 kDa) that is composed of a hexagonal-bilayer hemoglobin structure with a high oxygen (O_2_) binding capacity, carrying up to 156 O_2_ molecules (versus 4 for human hemoglobin) in a saturated state^30^. Owing to this exceptional O_2_-binding capacity, M101 releases O_2_ according to a simple gradient and possesses antioxidative properties. It also exhibits intrinsic superoxide dismutase (SOD)-like activity, preventing the occurrence of potentially harmful hemoglobin degradation products such as heme-protein-associated free radical species (ROS), or ROS related to ischemia in the solution^31,32^. These desirable properties of M101 open new vistas for its wide industrial and therapeutic applications. The efficacy of M101 used ex vivo or in vivo as an additive to preservation solutions for preventing ischemia/reperfusion injuries has already been demonstrated in preclinical studies in kidney^33^, heart^34^, pancreas^32^, lung^35^ and liver^36^. In the context of inflammation control, supplementation with M101 has shown reduced inflammation and fibrosis in kidney epithelium during static preservation^34^. Moreover, lung grafts preservation and perfusion with M101 decreased edema formation, apoptotic cell death and plasma levels of pro-inflammatory IL-6, hence, improving post-transplant lung function^37^. Furthermore, no immunogenic, allergenic or prothrombotic effects have been reported with in vivo administration^31^ or in the recently carried out first human trial of M101 in kidney transplantation^38^.

Recent advances have established M101 as a promising molecule that can be tested for its potential therapeutic efficacy, especially in the context of inflammatory diseases. This study explores the potential breakthrough role of M101 application for the treatment of periodontal inflammation, subsequently improving periodontal wound healing and tissue regeneration. In this study, the pro-healing effects of M101 on the *P. gingivalis*-induced inflammation have been investigated in vitro and in vivo as a proof of concept.

## Results

### Transmission electron microscopy

**Figure 1:**
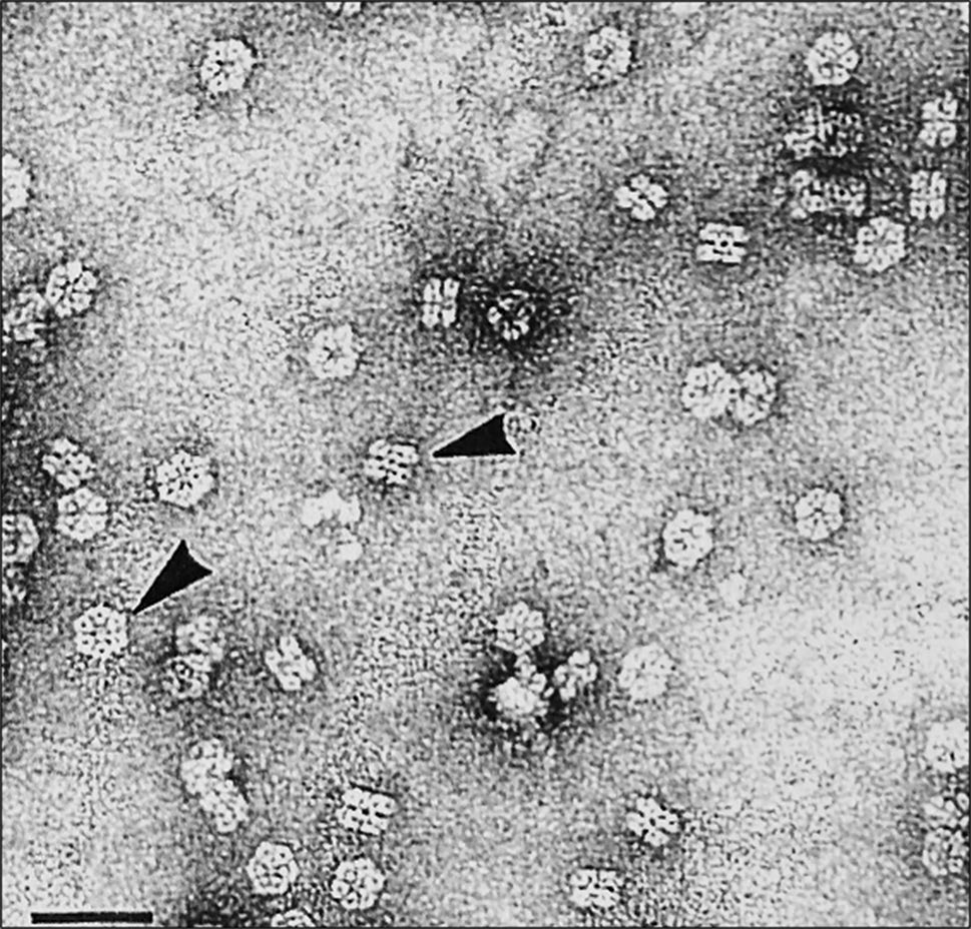
M101 structure. Electron micrographs of M101, negatively stained with 2% uranyl acetate, showing the two frequent orientations, face view and lateral view (arrows). Scale bar 60 nm.

M101 is an extracellular hexagonal-bilayer hemoglobin of high molecular weight (∼ 3,600 kDa), made by the association of 156 globin chains and 42 non-globin linker chains, making a quaternary structure of two overlapping hexagons of approximately 27 nm between parallel sides (face view) and 15 nm of thick (profile view) (Fig. 1).

### M101 is non-cytotoxic

**Figure 2:**
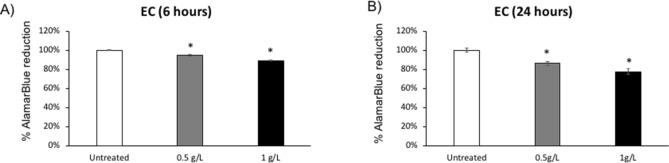
Cytocompatibility of M101. (**A**,**B**) Metabolic activity of human oral epithelial cells (EC) with exposure to M101 (0.5, and 1 g/L) at (**A**) 6 h, (**B**) 24 h. Data are expressed as the mean ± SD. *Difference versus untreated EC, p < 0.05. % Alamarblue reduction ≤ 20% is not considered cytotoxic.

To assess cytocompatibility of M101, M101 concentrations (0.5 and 1 g/L) were added to EC culture (Fig. 2). After 6 and 24 h of exposure to M101, the metabolic activity of EC was not reduced more than 10% and 20% respectively (Fig. 2). Thus, M101 concentrations up to 1 g/L were considered non-cytotoxic and an optimal dose of 1 g/L was selected for further experiments.

### M101 modulates release of key inflammatory mediators

#### M101 down-regulates release of pro-inflammatory mediators

**Figure 3:**
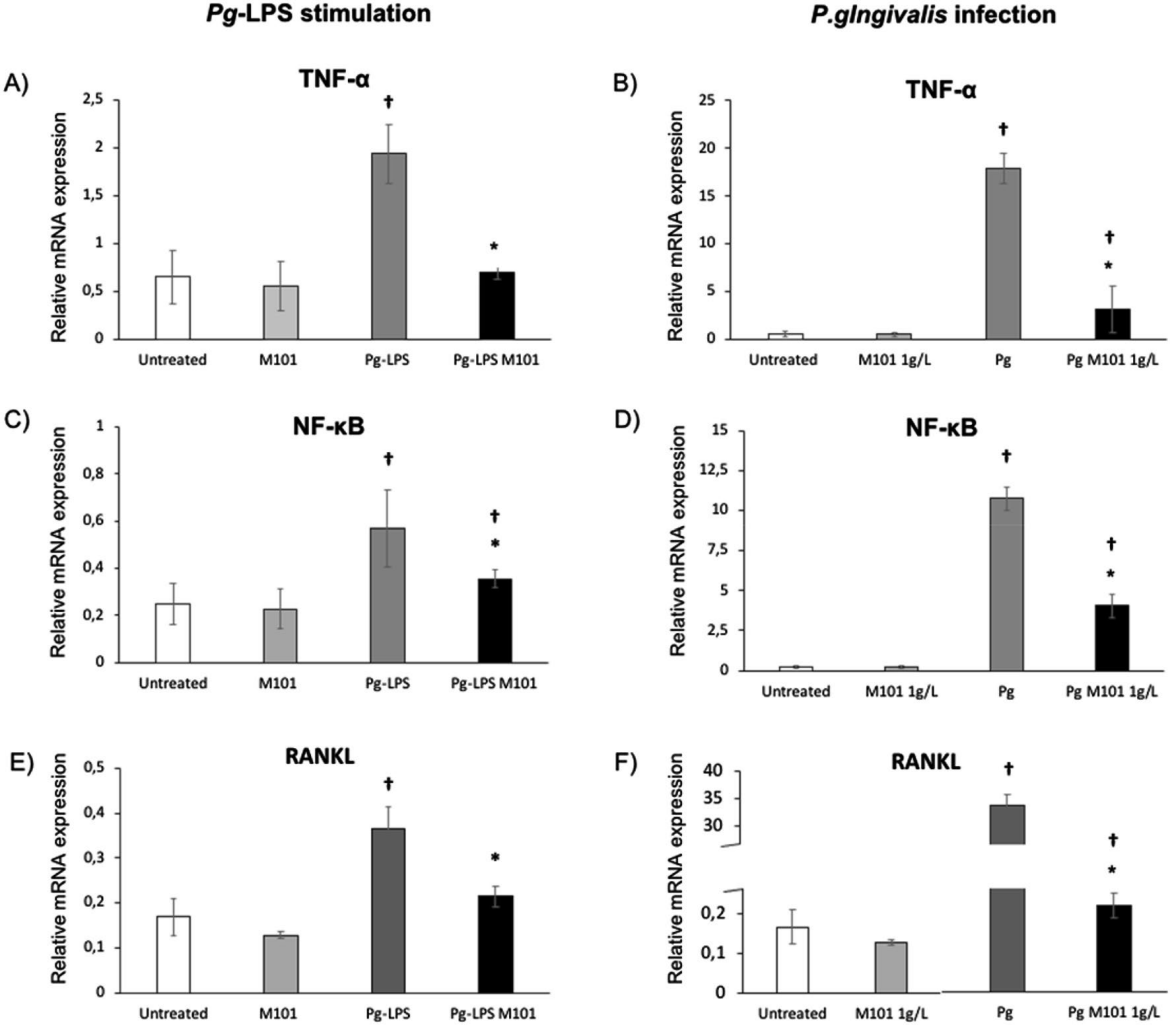
M101 reduces the gene expression of key pro-inflammatory markers in *P. gingivalis*-induced inflammation in EC. M101 treatment (1 g/L) for 6 h decreased the relative mRNA expression of pro-inflammatory markers in *Pg*-LPS (10 µg/mL) stimulated human gingival epithelial cells (EC). (**A**) TNF-α, (**C**) NF-κb, (**E**) RANKL and *Pg*-infected EC (MOI = 100). (**B**) TNF-α, (**D**) NF-κb, (**F**) RANKL. Data are expressed as the mean ± SD. †Difference versus untreated EC, p < 0.05, *difference versus *Pg*-LPS stimulated EC or *Pg*-infected EC, p < 0.05.

**Figure 4:**
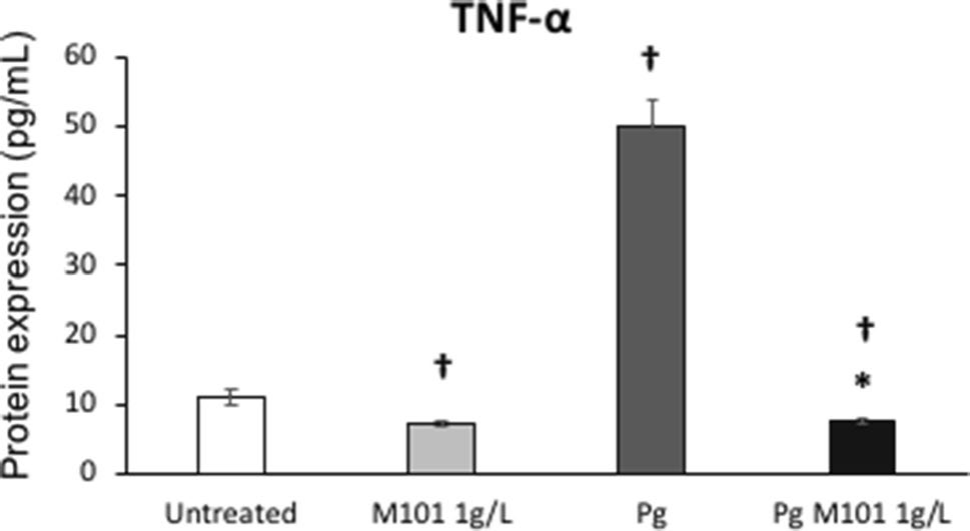
MI01 reduces the protein expression of TNF-α in *P. gingivalis*-induced inflammation in EC. M101 treatment (1 g/L) for 6 h decreased the protein expression of TNF-α in untreated human gingival epithelial cells (EC) and *Pg*-infected EC (MOI = 100). Data are expressed as the mean ± SD. ^†^Difference versus untreated EC, p < 0.05, *difference versus *Pg*-infected EC, p < 0.05.

To evaluate the potential anti-inflammatory effect of M101, EC were stimulated by lipopolysaccharide of *P. gingivalis* (*Pg*-LPS) (10 μg/mL) or infected with *P. gingivalis* at multiplicity of infection (MOI) of 100 for 24 h, and subsequently, treated with M101 for 6 h. As expected, *Pg*-LPS stimulation and *P. gingivalis* infection increased significantly the relative mRNA expression of key pro-inflammatory and pro-resorptive markers such as TNF-α, NF-κB and RANKL. For instance, TNF-α expression was increased by 3 folds upon stimulation with *Pg*-LPS, while 27 folds by *P. gingivalis* infection (Fig. 3A,B). For NF-κB expression, a 2.3 folds rise was observed with *Pg*-LPS stimulation and 43 folds by *P. gingivalis* infection of EC (Fig. 3C,D). Likewise, RANKL expression was up-regulated 2.2 folds and 200 folds by *Pg*-LPS and *P. gingivalis* respectively (*p* < 0.05) (Fig. 3E,F). Interestingly, this up-regulation of pro-inflammatory and pro-resorptive molecules’ expression in EC was reduced considerably with M101 treatment. TNF-α expression in EC exposed to *Pg*-LPS was reduced by 2.7 folds (63.5%) with M101 treatment (*p* < 0.05) (Fig. 3A). Similarly, M101 treatment of *P. gingivalis*-infected EC resulted in 5.7 folds (82.5%) decrease in TNF-α release (*p* < 0.05) (Fig. 3B). Furthermore, M101 down-regulated the NF-κB expression in EC exposed to *Pg*-LPS by 1.6 folds (37.6%) (*p* < 0.05) (Fig. 3C), while the same concentration of M101 resulted in 2.6 folds (62.1%) reduction of NF-κB release in *P. gingivalis*-infected EC (Fig. 3D). Besides, M101 treatment of *Pg*-LPS stimulated and *P. gingivalis*-infected EC minimized the RANKL expression by 0.6 folds (41.3%) and 2.1 folds (53.4%) respectively (*p* < 0.05) (Fig. 3E,F). To confirm the anti-inflammatory effect of M101 at protein level, an ELISA was carried out to determine the protein level of TNF-α in *P. gingivalis-*infected EC treated with M101 (Fig. 4). As expected, exposure of EC to *P. gingivalis* increased significantly the protein expression of TNF-α by 4.6 folds (*p* < 0.05). Notably, the TNF-α protein level in *P. gingivalis*-infected EC was reduced significantly (85%, 6.7 folds) by M101 (*p* < 0.05), confirming the results observed at the mRNA level.

**Figure 5:**
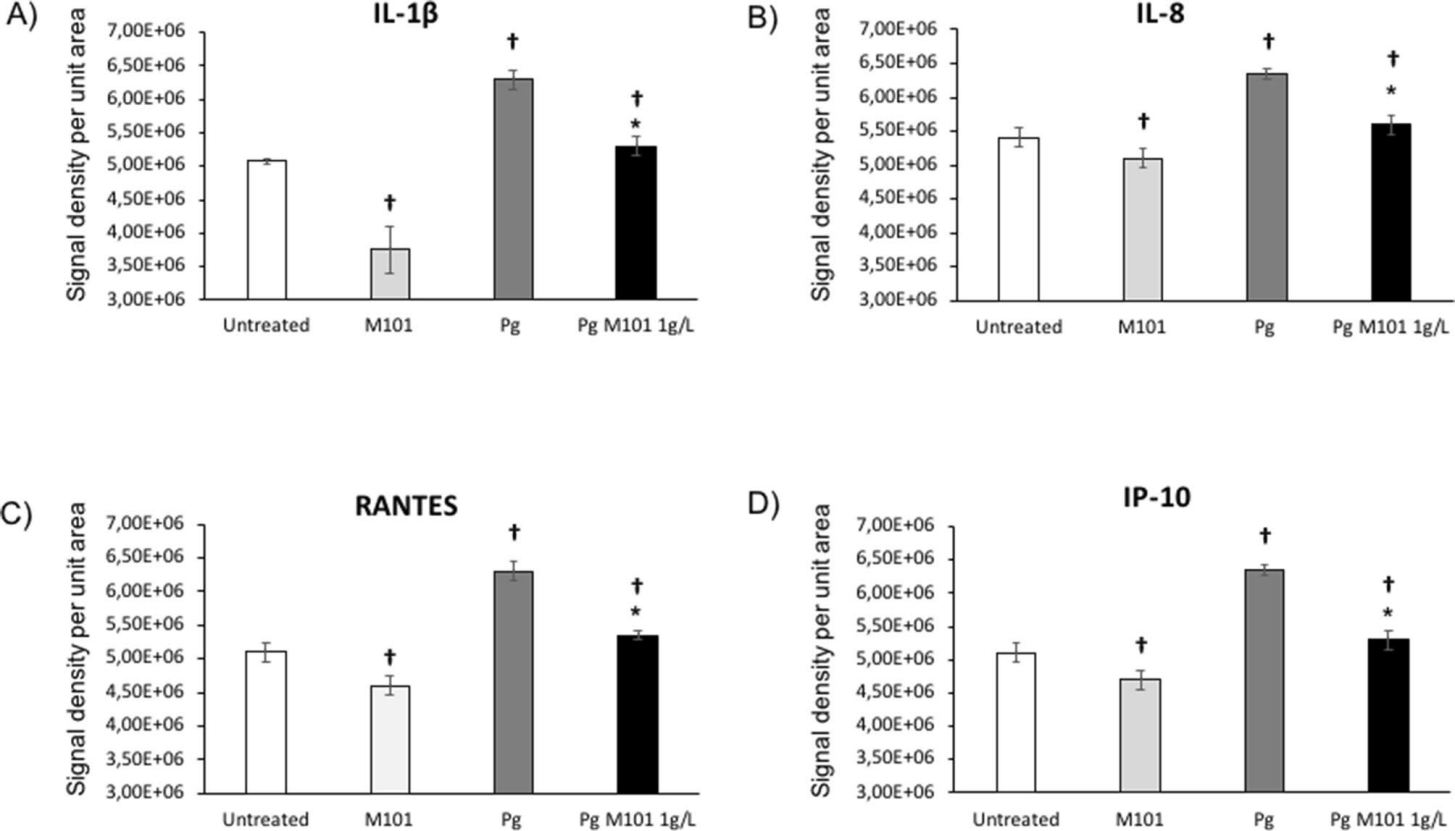
M101 reduces the protein expression of pro-inflammatory cytokines and chemokine ligands in *P. gingivalis*-induced inflammation in EC. The expression levels amongst untreated EC, M101-treated EC, *Pg*-infected EC and M101 treated *Pg*-infected EC were compared. M101 treatment (1 g/L) for 6 h decreased the protein expression of pro-inflammatory markers (**A**) IL-1β, (**B**) IL-8, (**C**) RANTES, (**D**) IP-10 in untreated and Pg-infected EC (MOI = 100). Data are expressed as the mean ± SD. ^†^Difference versus untreated EC, p < 0.05, *difference versus *Pg*-infected EC, p < 0.05.

Additionally, the proteomics array analysis corroborated our results as the protein expression of several other important pro-inflammatory mediators was also downregulated with M101 treatment. In particular, infection of EC by *P. gingivalis* markedly increased the levels of pro-inflammatory molecules including interleukins (IL-1β, IL-8), Regulated on Activation Normal T Cell Expressed and Secreted (RANTES) and interferon gamma receptor protein-10 (IP-10). However, significant reductions in IL-1β (26.1% versus untreated EC; 15.87% versus *P. gingivalis*-infected EC) (Fig. 5A), IL-8 (7.8%; 11.81%) (Fig. 5B), RANTES (9.8%; 15%) (Fig. 5C) and IP-10 (14.6%; 16.54%) (Fig. 5D) release in EC were observed after treatment with M101 (*p* < *0.05*) (Fig. 5).

#### M101 up-regulates release of anti-inflammatory mediators

**Figure 6:**
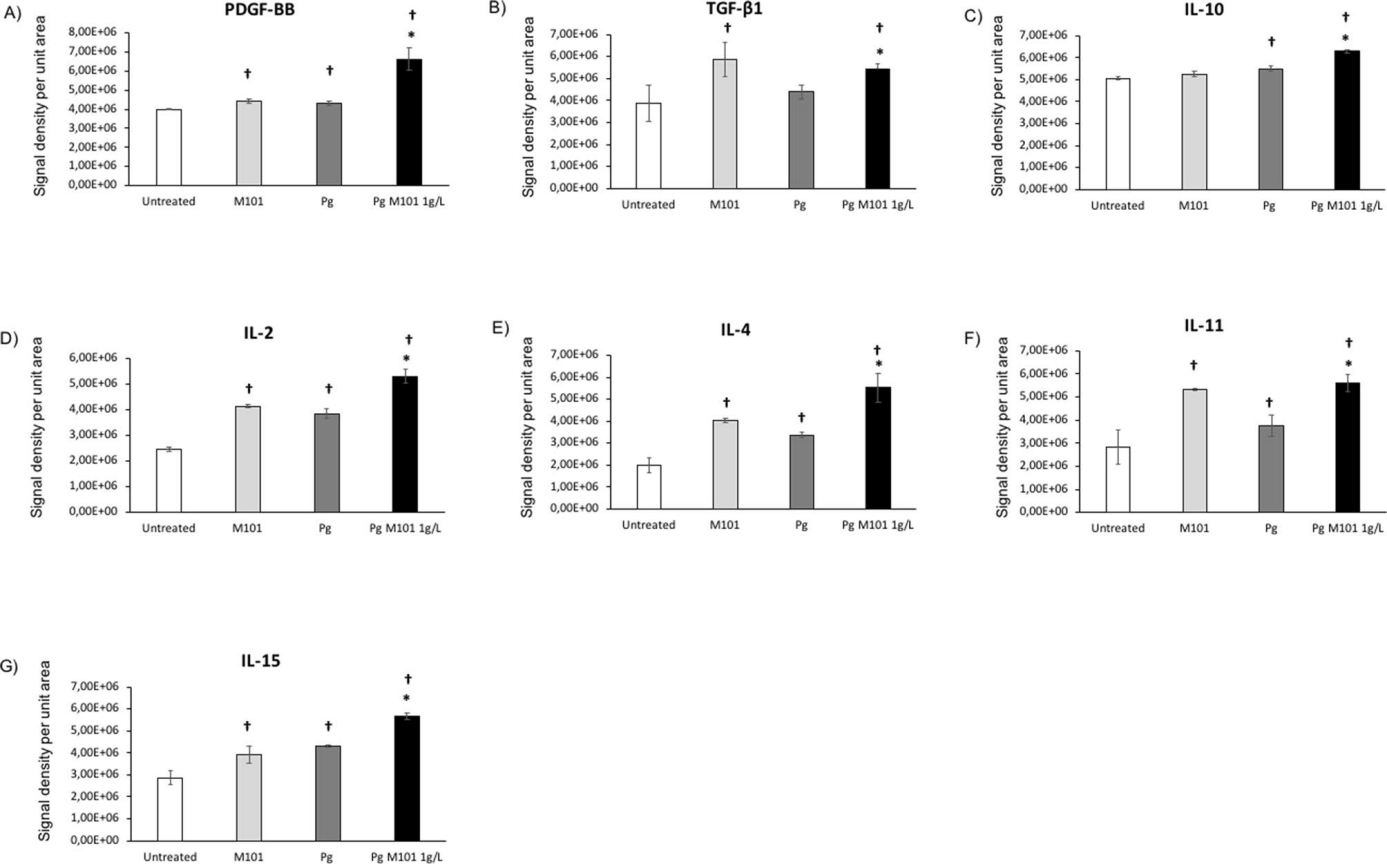
M101 promotes the protein expression of anti-inflammatory mediators in *P. gingivalis*-induced inflammation in EC. The expression levels amongst untreated EC, M101-treated EC, *Pg*-infected EC and M101 treated Pg-infected EC were compared. M101 treatment (1 g/L) for 6 h increased the protein expression of anti-inflammatory markers in human gingival epithelial cells (EC). (**A**) PDGF-BB, (**B**) TGF-β1, (**C**) IL-10, (**D**) IL-2, (**E**) IL-4, (**F**) IL-11, (**G**) IL-15 in untreated and *Pg*-infected EC (MOI = 100). Data are expressed as the mean ± SD. †Difference versus untreated EC, p < 0.05, *difference versus *Pg*-infected EC, p < 0.05.

Besides the downregulation of pro-inflammatory mediators’ release, the proteomics array confirmed that treatment with M101 resulted in significant upregulation of some anti-inflammatory markers such as PDGF-BB (10% versus untreated EC; 54.31% versus *P. gingivalis*-infected EC), TGF-β1 (51.36%; 23.64%) and IL-10 (4.2%; 14.36%), respectively (*p* < 0.05) (Fig. 6). In addition, M101 also modulated the expression of several other important interleukins such as IL-2 (67.7%; 37.82%), IL-4 (100%; 63.56%), IL-11 (88.5%; 49.27%) and IL-15 (36.5%; 32.28%) (*p* < 0.05) (Fig. 6).

#### M101 up-regulates release of extracellular matrix and immune modulators

**Figure 7:**
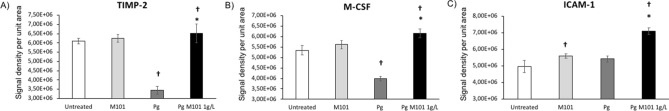
M101 promotes the release of ECM and immune modulators in *Pg*-induced inflammation in EC. The expression levels amongst untreated EC, M101-treated EC, *Pg*-infected EC and M101 treated *Pg*-infected EC were compared. M101 treatment (1 g/L) for 6 h increased the protein expression of (**A**) TIMP-2, (**B**) M-CSF and (**C**) ICAM-1 in untreated and *Pg*-infected EC (MOI = 100). Data are expressed as the mean ± SD. ^†^Difference versus untreated EC, p < 0.05, *difference versus *Pg*-infected EC, p < 0.05.

Treatment of EC with M101 resulted in a significant increase in the release of extracellular matrix (ECM) and immune modulators including Tissue inhibitor of metalloproteinases-2 (TIMP-2) (2.5% versus untreated EC; 89.83% versus *P. gingivalis*-infected EC), Macrophage colony-stimulating factor (M-CSF) (5%; 54.14%), and Intercellular Adhesion Molecule-1 (ICAM-1) (6.7%; 21.78%) (*p* < 0.05) (Fig. 7).

#### M101 up-regulates expression of pro-resolution mediator Resolvin-E1 receptor

**Figure 8:**
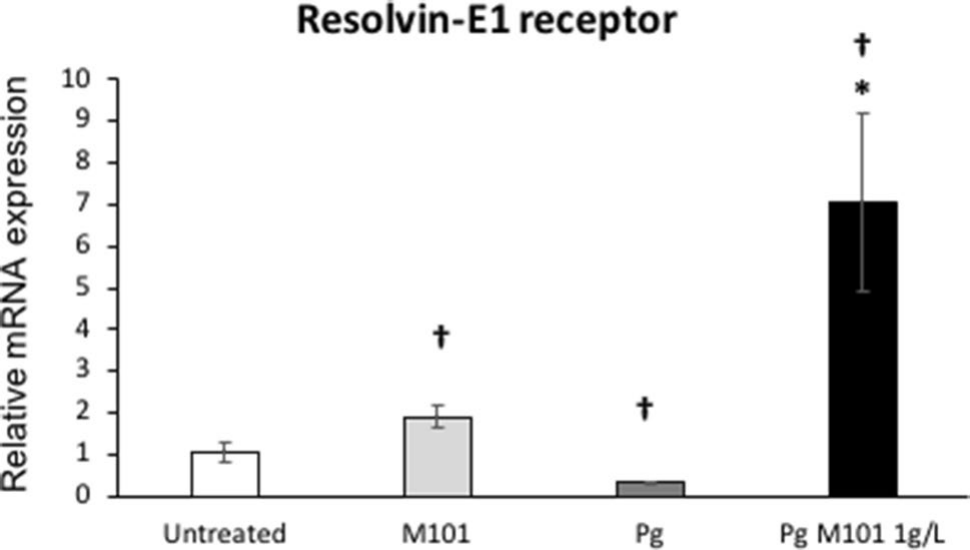
M101 up-regulates the gene expression of pro-resolutive Resolvin-E1 receptor in *P. gingivalis*-induced inflammation in EC. The expression levels amongst untreated EC, M101-treated EC, *Pg*-infected EC and M101 treated *Pg*-infected EC were compared. M101 treatment (1 g/L) for 6 h increased the relative mRNA expression of resolvin-E1 receptor in untreated and *Pg*-infected EC (MOI = 100). Data are expressed as the mean ± SD. ^†^Difference versus untreated EC, p < 0.05, *difference versus *Pg*-infected EC, p < 0.05.

To evaluate the potential pro-resolutive effect of M101, EC were infected with *P. gingivalis* (MOI = 100) for 24 h, and subsequently, treated with M101 for 6 h. *P. gingivalis* infection led to a significant decrease (3 folds) in the expression of resolvin-E1 receptor. However, M101 treatment showed an increase in its mRNA expression (1.8 folds versus untreated EC; 20 folds versus *P. gingivalis*-infected EC) (*p* < 0.05) (Fig. 8).

### *M101 reduced P. gingivalis growth *in vitro

**Figure 9:**
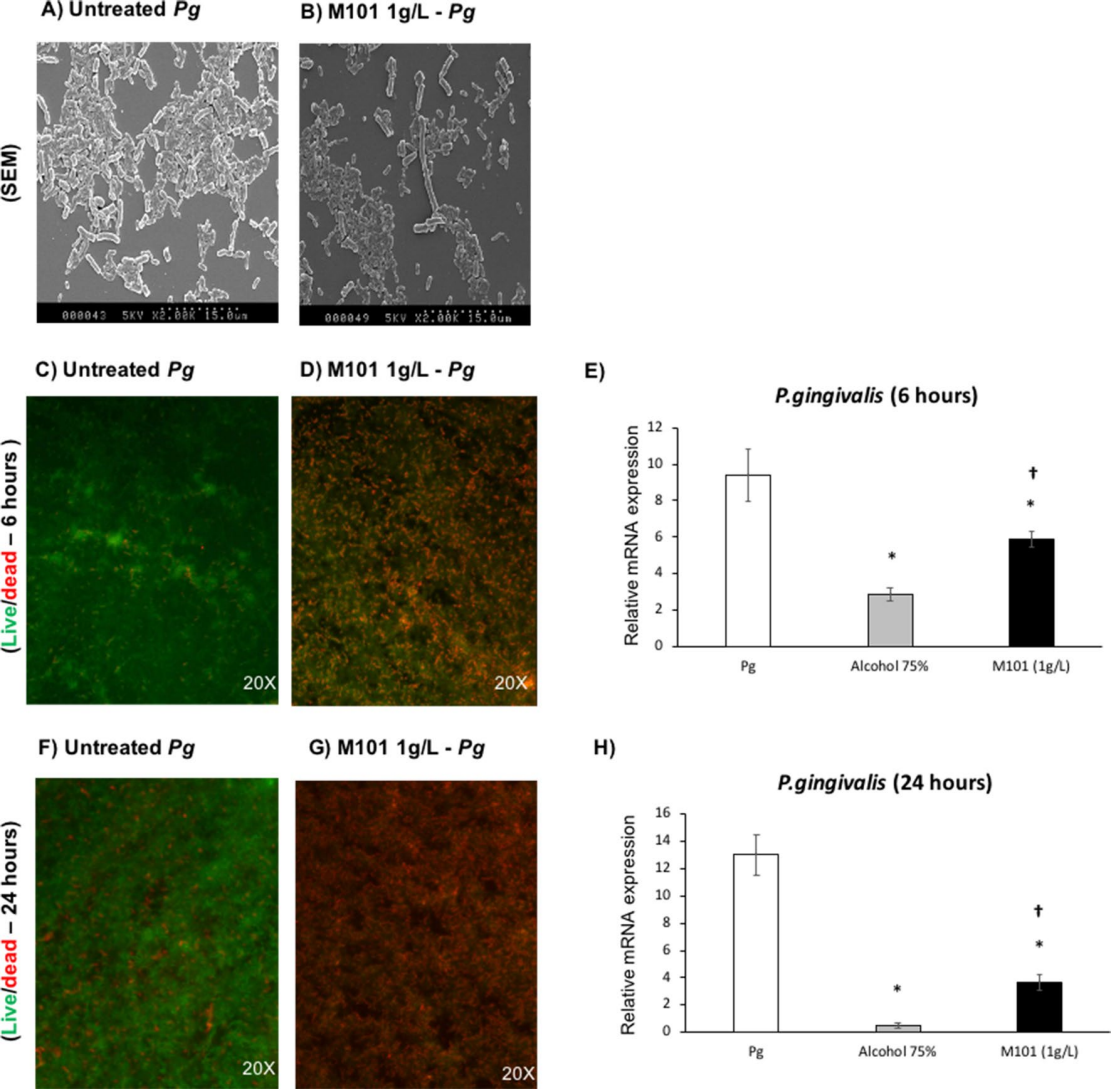
M101 reduces *P. gingivalis* growth in vitro. M101 treatment (1 g/L) decreased *P. gingivalis* (*Pg*) biofilm on glass surface; (**A**,**B**) scanning Electron Microscopy at 2 h (SEM) of (**A**) *Pg* biofilm, (**B**) M101-treated *Pg* biofilm (**C**,**D**) live/dead assay of *Pg* biofilm at 6 h (green = living *Pg*, red = dead *Pg*), (**E**) relative mRNA expression of *Pg* in *Pg* biofilm treated with M101 (1 g/L) for 6 h by RTqPCR, (**F**,**G**) live/dead assay of *Pg* biofilm at 24 h (green = living *Pg*, red = dead *Pg*), (**H**) relative mRNA expression of *Pg* in *Pg* biofilm treated with M101 (1 g/L) for 24 h by RTqPCR. Data are expressed as the mean ± SD. *Difference versus untreated *Pg*, p < 0.05, †difference versus alcohol 75%, p < 0.05.

To evaluate the potential impact of M101 treatment on *P. gingivalis* biofilm, *P. gingivalis* (1 × 10^8^ CFU) was grown over glass surface for 3 days. The obtained *P. gingivalis* biofilms were then treated with M101. SEM analysis revealed quantitative and qualitative changes in *P. gingivalis* growth and morphology (Fig. 9A,B). The bactericidal property of M101 was observed with Live/Dead staining as *P. gingivalis* biofilm treated with M101 at 6 and 24 h demonstrated a notable reduction in the living *P. gingivalis* concomitant with an increase of dead cells compared to the untreated control (Fig. 9C,D,F,G). This antibacterial effect was further corroborated with a significantly down-regulated 16s rRNA expression of *P. gingivalis* in M101-treated *P. gingivalis* biofilms at 6 h (1.6 folds) and 24 h (3.5 folds) compared to that measured in the untreated controls (*p* < 0.05) (Fig. 9E,H).

### *M101 reduced P. gingivalis-induced subcutaneous inflammatory lesion *in vivo

**Figure 10:**
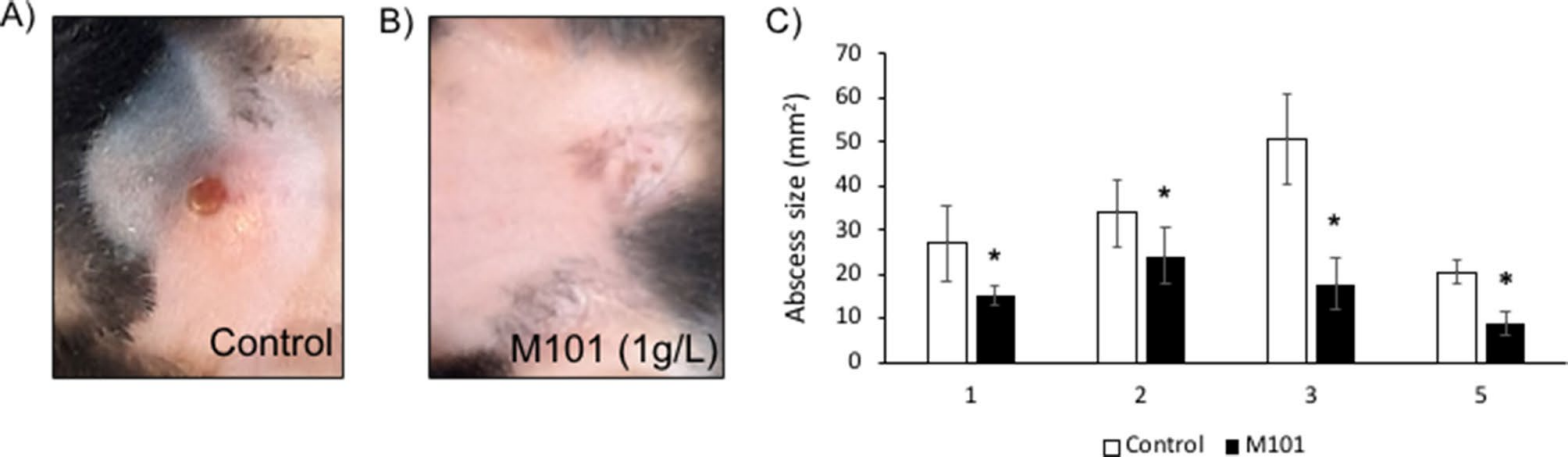
M101 promotes healing in *P. gingivalis*-induced inflammation in vivo. (**A**) Untreated control lesion on day 1, (**B**) M101 (1 g/L)-treated lesion on day 1, (**C**) abscess size (mm^2^) of untreated and M101 (1 g/L) treated lesion during follow-up (n = 5/group). Data are expressed as the mean ± SD. *Difference versus untreated subcutaneous inflammatory calvarial lesion size, p < 0.05.

**Figure 11:**
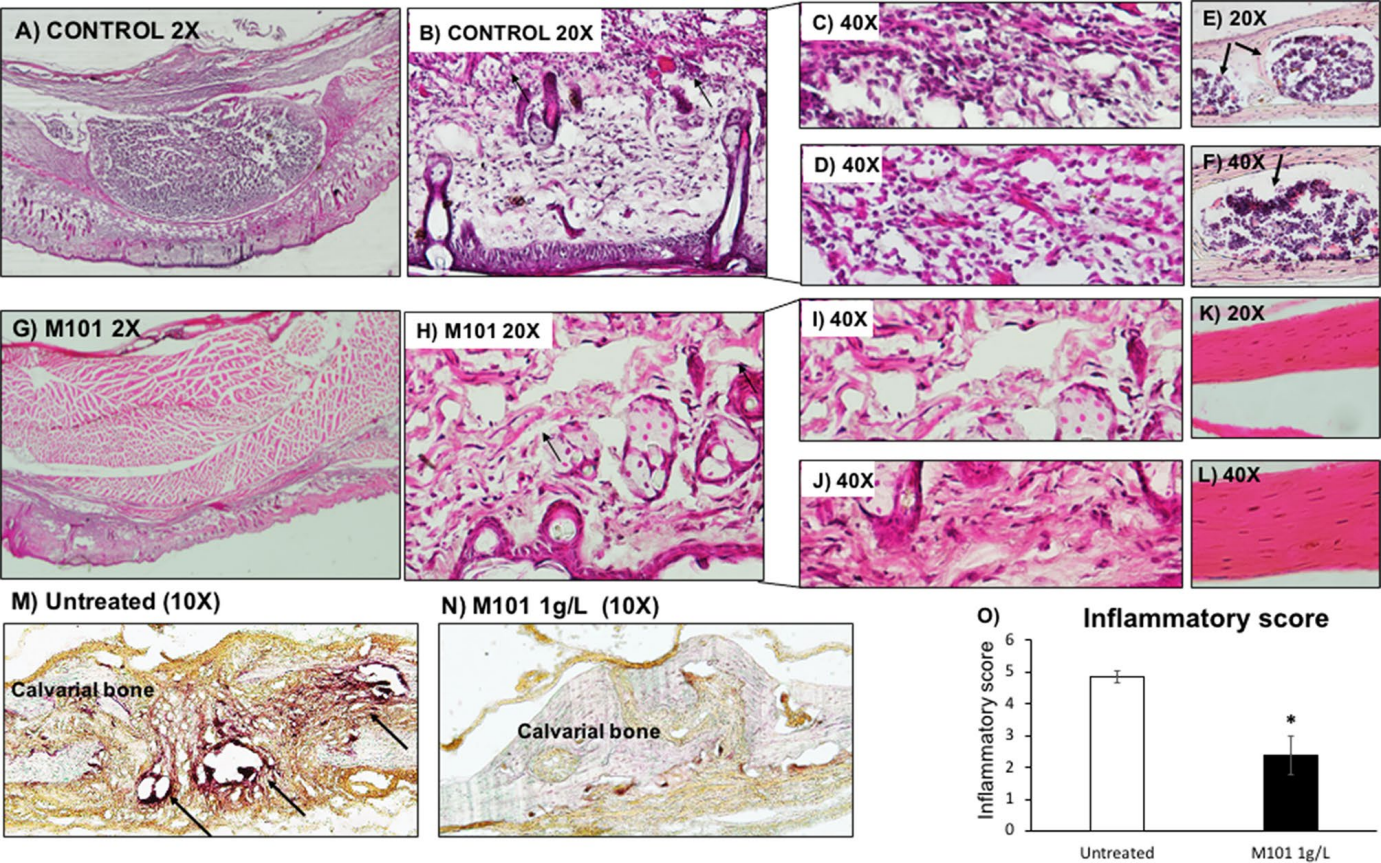
M101 reduces *P. gingivalis*-induced inflammation-mediated bone resorption in vivo. Inflammatory score assessment on histological sections of *Pg*-induced subcutaneous lesion (**A**) untreated control ×2, (**B**) soft tissue ×20, (**C**,**D**) soft tissue ×40, (**E**) calvarial bone ×20, (**F**) calvarial bone ×40, (**G**) lesion treated by M101 (1 g/L) ×2, (**H**) soft tissue ×20, (**I**,**J**) soft tissue ×40, (**K**) calvarial bone ×20, (**L**) calvarial bone ×40, (**M**) untreated control ×10 (TRAP assay), (**N**) M101-treated lesion ×10 (TRAP assay), (**O**) inflammatory score. Data are expressed as the mean ± SD. *Difference versus untreated subcutaneous inflammatory calvarial lesions’ inflammatory score, p < 0.05.

To evaluate the anti-inflammatory effects of M101 in vivo, a calvarial subcutaneous abscess was induced by *P. gingivalis* injection in mice. Clinical assessment of untreated and M101-treated (1 g/L) subcutaneous inflammatory calvarial lesions in mice was carried out by measuring the clinical abscess size over a period of 5 days (Fig. 10A,B). M101-treated mice demonstrated a significantly decreased abscess size, on average 50.23%, when compared to the untreated control mice (*p* < 0.05) (Fig. 10C). Histomorphometric analysis of mice with *P. gingivalis*-induced subcutaneous inflammatory calvarial lesion treated with M101 exhibited an appreciably reduced inflammatory cell infiltrate in soft tissue compartment (derma/subderma) (Fig. 11G–J) compared to the untreated controls (Fig. 11A–D). Besides, the M101-treated mice showed a significantly decreased (50.86%) inflammatory score in comparison with the untreated controls during follow-up (*p* < 0.05) (Fig. 11O). The quantity and quality of bone was improved with a smaller number of lacunae in M101-treated mice compared to that of the control mice (Fig. 11E,F,K,L). In the context of inflammation-mediated resorptive activity in calvarial bone, TRAP assay also demonstrated a greater inflammatory and osteoclastic activity in untreated controls (Fig. 11M) in comparison with the M101-treated mice (Fig. 11N).

## Discussion

The control of inflammation and infection is crucial for achieving an optimal wound healing response in the context of periodontitis management. In this study, M101, an oxygen transporter derived from *Arenicola marina,* was tested for its anti-inflammatory and anti-infectious potential based on its anti-oxidative and tissue oxygenation properties.

M101 is a natural biopolymer demonstrating unique intrinsic traits of therapeutic potential such as its high oxygen affinity and SOD‐like activity linked to Cu/Zn^31^. Direct oxygenation of damaged tissue is a therapeutic strategy paving way for the use of oxygen carriers such as M101. Indeed, preservation and perfusion of rat and human pancreas (ex vivo)^32^ and porcine kidneys (in vivo)^33^ with M101 solution improved graft function and transplantation outcome. Interestingly, first M101 use in human clinical trial demonstrated that kidney preservation with M101 solution (1 g/L) before transplantation, improved graft survival and renal function. No immunological/ allergic reactions or infections were reported confirming its absence of cytotoxicity and immunogenicity^38^.

This study establishes the cytocompatibility of M101 (1 g/L) in EC as they represent the first line of defense in the periodontium against any foreign aggression^12^. The same was further corroborated in FB, OB and BALB/3T3 cells (Supplementary Figs. S1–S3). The selected concentration used in this study (1 g/L) has already demonstrated positive outcomes in vivo and in clinical trials^33,38^.

Elevated levels of pro-inflammatory mediators, such as IL-1β, TNF-α, IL-8 and RANKL, have been associated with periodontal inflammation^39–41^. Increased expression of pro-inflammatory mediators such as IL-1β, IL-8, TNF-α and RANKL in *P. gingivalis*-infected EC has been well-documented^42–44^ and their decrease has been correlated with periodontal healing^45^. Besides the notorious pro-inflammatory cytokines, certain chemokine ligands such as IP-10^46^ and RANTES^47^ have also been associated with periodontitis. RANTES causes activation and chemotaxis of leukocytes inducing the release of other cell mediators, thus, exacerbating periodontal inflammation. In the context of inflammation-mediated bone resorption, IP-10 promotes osteoclastogenesis owing to its involvement in leukocyte and osteoclast precursor diapedesis, and its increase causes inflammation-mediated tissue damage via RANKL, causing bone resorption in periodontitis^46,48,49^. These classical pro-inflammatory markers of periodontitis present potential therapeutic target for periodontitis treatment^50,51^. In this regard, several studies demonstrated that pharmacological treatments, including the use of synthetic drugs and natural compounds, such as TNF-α antagonist^52^, angiotensin II receptor blocker^53^, non-selective β-blocker^54^ or anti-RANKL antibodies^55^, resulted in reduced levels of TNF-α, NF-κB and RANKL, thereby, resolving experimental periodontitis in vivo*.* The use of such aforementioned conventional pharmacological drugs in periodontal therapy is not devoid of side effects, thus, necessitating the development of novel therapeutic agents and strategies to provide safer alternatives.

Our results demonstrate that M101 reduced significantly the release of TNF-α, NF-κB, RANKL, IL-1β and IL-8 from *P. gingivalis*-infected EC indicating a decrease in inflammation. Regarding pro-inflammatory chemokine ligands, an intensification of RANTES and IP-10 release occurred with *P. gingivalis* infection in EC as shown previously for EC predominant EC-macrophage co-culture^44^ and macrophages^56^. However, M101 decreased this exacerbated RANTES response in *P. gingivalis*-infected EC. Similar trend of results was seen in *P. gingivalis*-infected macrophages treated with an anti-inflammatory kavain analogue^57^. Moreover, IP-10 release from *P. gingivalis*-infected EC was down-regulated with M101 treatment.

The importance of pro-healing molecules in resolution of inflammation and optimal wound healing cannot be overstated^58^. For instance, PDGF promotes tissue repair and regeneration^59,60^. TGF-β isoforms play an important role in EC proliferation and differentiation, and regulation of their barrier function^61^, thus promoting wound healing^62^. IL-10, one of the most potent anti-inflammatory cytokine, is considered vital for regenerative wound healing as it downregulates the production of several pro-inflammatory mediators and prevents bone resorption^63–66^. Interestingly, the protein expression of these pro-healing molecules (PDGF-BB, TGF-β1 and IL-10) was significantly increased by M101 treatment of *P. gingivalis*-infected EC. Furthermore, IL-2^67,68^, IL-4^69^, IL-11^70–72^, IL-15^73,74^ play an important role in counteracting inflammation and infection. Notably, this study demonstrated an increase in IL-2, 4, 11 and 15 in untreated EC and *P. gingivalis*-infected EC treated with M101.

The extracellular matrix and immune modulators such as TIMP-2^75,76^, M-CSF^77,78^ and ICAM-1^79,80^ also contribute towards host-immune response and enhance bacterial clearance. M101 treatment of *P. gingivalis*-infected EC significantly increased TIMP-2, ICAM-1 and M-CSF release, hence, promoting bacterial clearance and wound healing. Pro-resolutive mediators such as resolvin-E1 play a role is an active regulation of healing and regeneration of the damaged tissues rather than a mere passive termination of the inflammatory process^21^. Resolvin-E1 rescues periodontal tissues from inflammation and osteoclast-mediated degradation by reversing NF-κB activation and dysbiosis, thus, promoting tissue homeostasis in experimental periodontitis^81^. M101 treatment resulted in a remarkable increase in resolvin E1 receptor expression in *P. gingivalis*-infected EC.

Bacterial persistence in the periodontal pockets leads to an accumulation of inflammatory cell infiltrate and consequent high proteolytic enzymes’ content that can hinder the periodontal regenerative process by destabilizing the clot and formation of long junctional epithelium. The beneficial effects of M101 on EC in the periodontium would be explained by M101’s note-worthy oxygenation capacity in wound environments and periodontal pockets where oxygen improves vascularization and counteracts infection and edema, leading to improved wound healing and regenerative response^82,83^. Nonetheless, the underlying pathways impacted by M101 should be further explored.

In addition to the inflammation modulating property, M101 also demonstrated an anti-bacterial effect on *P. gingivalis* biofilm. In this context, M101 treatment showed a significant reduction in *P. gingivalis* growth assessed through SEM and Live/dead assay. SEM images also revealed a marked change in the morphology of *P. gingivalis* treated with M101. The M101-treated bacteria appeared notably longer (even up to 3 folds). This change in bacterial morphology could possibly be related with the oxygen carrying capacity of M101 which causes oxygen stress leading to their abnormal cell division and eventual death^84^. Additionally, bacterial proteins can also be oxidized and damaged during oxidative stress or due to deleterious effects of ROS, causing severe impairment of function and bacterial cell death^85^. M101’s anti-bacterial effect was further confirmed by a decrease in *P. gingivalis’* relative gene expression in M101-treated *P. gingivalis* biofilms in comparison to the untreated controls. However, further studies should be carried out with *P. gingivalis* and other periodontal pathogens to fully establish the anti-infectious trait of M101.

R- and K-gingipain proteases of *P. gingivalis* can cause proteolysis of human hemoglobin to form heme by erythrolysis, however, oxyhemoglobin is refractory towards K-gingipains resulting in the formation of a hemoglobin hemachrome which is relatively stable towards their further attack. Moreover, R-specific gingipains show little degradative activity towards hemoglobin, although they can attack the oxyhemoglobin molecule forming methemoglobin^86,87^. Therefore, the higher molecular weight, extraordinary oxygen carrying capacity and intrinsic SOD-like activity of M101 in comparison to the human hemoglobin render protection to the M101 hemoglobin against proteolysis by gingipains, thus keeping it active against *P. gingivalis.* Nevertheless, further investigation into the interaction between *P. gingivalis* and M101 is warranted.

To validate these observed results in vivo, M101 was used to treat a mouse model of *P. gingivalis*-induced subcutaneous abscess at calvaria where M101 injection at the lesion site promoted wound healing. Inoculation of *P. gingivalis* at calvarial site induces formation of an abscess, inflammation of soft tissues, and destruction of the underlying bone^88^. Despite not being a true experimental periodontitis model, this presents a convenient and quick experimental model to simulate infection triggered-inflammation-mediated soft tissue and bone degradation. Histomorphometrically, the overall inflammatory score was significantly lower in mice who received M101 treatment following the formation of *P. gingivalis*-induced subcutaneous calvarial lesions. In this regard, M101 treatment significantly reduced the inflammatory cell infiltration (ICI) and subsequent soft tissue damage in comparison with the control mice. Furthermore, the bone healing was improved with fewer lacunae in M101-treated mice. TRAP assay also demonstrated greater osteoclastic activity in control mice compared to those treated with M101. Similar pattern of reduced inflammation and tissue damage has also been demonstrated using the same model of *P. gingivalis*-induced subcutaneous calvarial lesion treated with other potential pharmacological therapeutic agents such as miRNA (mmu-miR-155-5p)^89^, anti-inflammatory kavain analogue^57^ and probiotic^90^.

Taken together, the anti-inflammatory and anti-bacterial properties of M101 confirm its pro-healing effects that facilitate the recovery and regeneration of damaged tissues. Such pro-healing and pro-regenerative effects of M101 treatment may be correlated to the observed effects in vitro such as the decrease of pro-inflammatory markers and the increase in anti-inflammatory and pro-regenerative mediators; however, further studies are required to determine precisely the effect of a longer M101 treatment both in vitro and in vivo*.* Testing M101’s potential in other in vivo models, more relevant to periodontitis, could be instrumental in establishing M101 as a potential therapeutic agent in the context of periodontal wound healing and regeneration.

## Materials and methods

### Cell culture

Human oral epithelial cells (EC) (TERT-2 OKF-6, BWH Cell Culture and Microscopy Core, Boston, MA, USA) were cultured in Keratinocyte-SFM medium (Life Technologies, Saint-Aubin, France). To reduce the risk of contamination, 100 units/mL of penicillin and 100 μg/mL of streptomycin (PromoCell, Heildelberg, Germany) were added to all cell media. Cells were grown at 37 °C in a humidified atmosphere with 5% CO_2_ and media were changed each 3 days.

### Bacterial culture

The *P. gingivalis* strain (ATCC 33277) was purchased from the ATCC and cultured as described previously^13^. Briefly, bacterial culture was performed under strict anaerobic conditions at 37 °C in brain–heart infusion medium (BHI) (Sigma) supplemented with hemin (5 μg/mL) and menadione (1 μg/mL). For each experiment, bacteria were grown for 3 days. Before use, the bacterial culture was centrifuged and bacteria were washed twice with phosphate buffer saline (PBS). Ultrapure lipopolysaccharide of *P. gingivalis* (*Pg*-LPS) was purchased from Invivogen (Toulouse, France).

### Transmission electron microscopy

M101 was diluted (1:900) with saline buffer and applied to a very thin carbon substrate supported on a microgrid, stained with 2% (mass/volume) uranyl acetate solution as described earlier^31^. The specimens were examined with an electron energy of 80 keV, using a Jeol JEM-1200EX microscope.

### Metabolic activity assay

To assess the effect of different concentrations of M101 on EC metabolic activity, an AlamarBlue assay (Life Technologies, Saint-Aubin, France) was performed as described previously^27^. Twenty-four hours before the experiment, 2 × 10^5^ EC were seeded in each well of a 24-well plate. On the day of the experiment, cells were washed twice with PBS. 20 μL of M101 stock solution (50 g/L) was added to each well of a 24-well plate with 1 mL volume per well (final concentration of M101 as 1 g/L). Likewise, volumes of M101 stock solution were adapted to reach final concentration of 0.5 g/L. After treating EC with a range of M101 concentrations (0.5 g/L and 1 g/L) for 6 and 24 h, 200 µL of incubation media were transferred separately to a 96-well plate and absorbance was measured at OD_570_ and OD_595_ nm by a spectrophotometer (Multiskan, ThermoScientific) in order to determine the percentage of AlamarBlue reduction.

### Real time quantitative PCR

Twenty-four hours before the experiment, 2 × 10^5^ EC were seeded in each well of a 24-well plate. On the day of the experiment, cells were washed twice with PBS and exposed to *Pg*-LPS stimulation at a concentration of 10 μg/mL or infected with *P. gingivalis* at MOI = 100. After 24 h, the non-infected, *Pg*-LPS stimulated and infected EC were treated with M101 (1 g/L) for 6 h. Cell lysates were collected after Trizol extraction (Thermofisher Scientific).

To quantify relative mRNA expression, qPCR was performed on the cDNA samples. PCR amplification and analysis were performed with CFX Connect Real-Time PCR Detection System (Biorad, Miltry-Mory, France). Amplification reactions were performed using iTAq Universal SYBR Green Supermix (Biorad). Beta-actin (β-actin) was used as endogenous RNA control (housekeeping gene) in all samples obtained from cell lysates.

*Porphyromonas gingivalis* biofilm (1 × 10^8^ CFU/mL) was grown over glass discs in strict anaerobic environment at 37 °C for 3 days. The biofilm was gently washed with PBS twice and later, treated with M101 diluted in BHI medium for 6 and 24 h. Alcohol 75% was used as positive control. Samples from *P. gingivalis* biofilms (bacterial lysates) were collected with Trizol extraction and RT-qPCR was performed as described earlier^25^. For *P. gingivalis* biofilms analysis, universal bacterial sequence-2 (Universal BS2) was used as housekeeping gene. Normalized gene expression level with reference to the housekeeping gene was calculated. Primer sequences (β-actin, TNF-α, NF-kB, RANKL, Resolvin-E1 receptor, Universal BS2, *P. gingivalis* 16 s rRNA) were purchased from ThermoFischer Scientific (Table 1).

**Table 1:** Primer sequences.

Primer name	Primer sequence
β-Actin	5′AACGGCTCCGGCATGTGCAA3′ 3′CTTCTGACCCATGCCCACCA5′
TNF-α	5′AGGCGCTCCCCAAGAAGACA3′ 3′TCCTTGGCAAAACTGCACCT5′
NF-kB	5′GCGCATCCAGACCCACAATAAC3′ 3′GCCGAAGCTGCATGGACACT5′
RANKL	5′GCTCAACAAGGACACAGTGTGC3′ 3′CGCATCGGATTTCTCTGTCCCA5′
Resolvin-E1 receptor	3′ATAGAATGGAGGATGAAGATTACAACACT5′ 5′TCCCGAGGAAGCAGACGATG3′
Universal BS2	5′CCATGAAGTCGGAATCGCTAG3′ 3′GCTTGACGGGCGGTGT5′
*P. gingivalis* 16S rRNA gene	5′TACCCATCGTCGCCTTGGT3′ 3′CGGACTAAAACCGCATACACTTG5′

### ELISA

To evaluate the anti-inflammatory effect of M101, 2 × 10^5^ EC infected with *P. gingivalis* (MOI = 100) were treated with M101 for 6 h. Cell supernatants were collected and subjected to ELISA for the detection of TNF-α concentrations using Quantikine ELISA Human TNF-α kit (R&D systems, Abingdon, United Kingdom). ELISA immunoreactivity was quantified using a microplate reader at OD_450_ nm and the standard curve was used to calculate and compare the concentrations of TNF-α protein (pg/mL) in the samples.

### Proteomics array

To evaluate the impact of M101 treatment on the protein expression of cytokines in EC infected by *P. gingivalis* (MOI = 100), a Human Inflammation Antibody Array—Membrane (Abcam ab134003, Paris, France) was used according to manufacturer’s instructions. Prior to the experiments, 2 × 10^5^ EC previously infected with *P. gingivalis* MOI = 100 were treated with M101 at 1 g/L for 6 h. Then, supernatants were collected and subjected to proteomics analysis (see Supplemental File).

### Effect on bacterial growth over time

*Porphyromonas gingivalis *biofilm (1 × 10^8^ CFU/mL) was grown on glass discs in strict anaerobic environment at 37 °C. After 3 days of bacterial growth, 20 μL of M101 stock solution (50 g/L) was added to each well of a 24-well plate with 1 mL volume per well (making the final concentration of M101 as 1 g/L in each well) for 6 and 24 h. The untreated and M101-treated biofilms were prepared using a fluorescence-based LIVE/DEAD Cell Imaging Kit, Molecular Probe (Invitrogen, Thermofisher Scientific) for visualization under a fluorescence microscope (Leica DM4000B, France).

### Scanning electron microscopy (SEM)

Treated and untreated biofilms were dehydrated in a series of alcohol solutions (50, 70, 90, 100%), fixed with a mixture of sodium cacodylate and glutaraldehyde and sputter-coated for 7–15 min with platinum using spray coating Technics Hummer II (Technics, Alexandria, VA, USA). Images were captured using a scanning electron microscope Leica Cambridge Stereoscan 360 FE (Leica Cambridge Co., Cambridge, UK) and the software EDS 2006 (IXRF Systems Inc., Houston, TX, USA).

### P. gingivalis-induced subcutaneous calvarial abscess mouse model

Ten (n = 5/group) 8 weeks old male C57BL/6J mice (Charles River, L’Arbresle, France) were used in this study. All animals were regularly fed and kept in separate cages. All experimental protocols were approved by the Ethics Committee of Strasbourg named “Comité Régional d’Ethique en Matière d’Expérimentation Animale de Strasbourg (CREMEAS)” (APAFIS#23885-2020013114423121 v3) and followed relevant guidelines of the same. Mice were examined to evaluate pain and stress, and their weights were monitored daily. After anaesthesia, *P. gingivalis* (5 × 10^8^ CFU in 100 μL) was injected on calvaria subcutaneously to induce an abscess as described previously^89,90^ After 6 h, 100 μL of M101 (1 g/L) was subcutaneously injected in the test group while the control group was left untreated. The lesion size was measured each day for 5 consecutive days. Clinical abscess size measurements were carried out and comparison between control and test groups was done with Image J software, 1.46r (National Institute of Mental health (NIMH), Bethesda, Maryland, USA).

### Tissue preparation

After 5 days, mice were euthanized with an overdose of anesthesia followed by cervical dislocation. Afterwards, sections of calvarial bone along with skin were dissected and post-fixed by immersion overnight at 4 °C in a solution containing 4% paraformaldehyde in PBS (pH 7.4). After extensive washing in PBS, the samples were embedded in OCT (TissueTek, Sigma). Ten μm thick serial frontal sections of the calvarial samples were cut with a cryostat (Leica RM 2165, France). These 10 μm thick OCT embedded sections were dehydrated in xylene and rehydrated through a series of graded ethanol and stained with hematoxylin and eosin (H/E). Later, the stained sections were visualized and photographed with a microscope (Leica DM4000B, France) for further assessment.

### Histomorphometric analysis

To quantify the histological sections, samples were evaluated by double-blinded examiners as described earlier^83^. Inflammatory cell infiltration (ICI) was scored on a scale of 0 through 5, with 0 corresponding to no sign of inflammation, 1 corresponding to incipient ICI in the derma/subderma, 2 corresponding to mild ICI in the derma/subderma, 3 corresponding to moderate ICI in the derma/subderma and scarce inflammation in the surrounding bone, 4 corresponding to advanced ICI in the derma/subderma and mild inflammation in the surrounding bone, and 5 corresponding to severe ICI in the derma/subderma and advanced inflammation in the surrounding bone.

To analyze the inflammation-mediated osteoclastic activity, a tartrate-resistance acid phosphatase (TRAP) assay was performed with TRAP Kit (387A-1KT, Merck, France). The TRAP solution was prepared according to manufacturer’s instructions. The histological slides were immersed into freshly prepared TRAP solution and kept for 1 h at 37 °C avoiding any exposure to light. Subsequently, the slides were washed with deioinized water and rinsed with tap water before observing under the microscope (Leica DM4000B, France).

### Statistical analysis

All experiments were repeated at least three times in triplicates (technical and biological replicates) and statistical analysis was performed using ANOVA and Mann–Whitney U test. A *p* value < 0.05 was considered significant.

## Supplementary information

Supplementary information
