# Supplementary material for: A therapeutic oxygen carrier isolated from Arenicola marina decreased P. gingivalis induced inflammation and tissue destruction
Source: Sci Rep. 2020 Sep 8;10:14745. doi: 10.1038/s41598-020-71593-8 (PMC7479608; doi:10.1038/s41598-020-71593-8)
Supplement: Supplementary file 1 — Supplementary information [file 41598_2020_71593_MOESM1_ESM.pdf]

A therapeutic oxygen carrier isolated from Arenicola marina decreased P.gingivalis induced inflammation and tissue destruction

Fareeha Batool, Céline Stutz, Catherine Petit, Nadia Benkirane-Jessel, Eric Delpy, Franck Zal, Elizabeth Leize-Zal, Olivier Huck

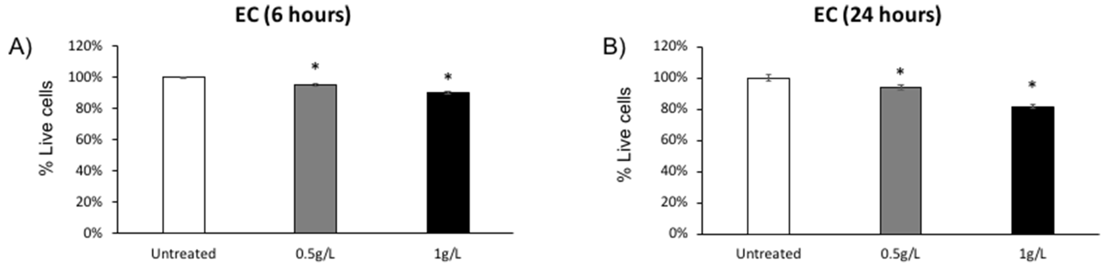

Supplementary figure 1: Percentage live Oral Epithelial cells (EC) with exposure to M101 (0.5, and 1g/L) at A) 6 hours B) 24 hours. Data are expressed as the mean ± SD. * Difference versus untreated EC, $p<0.05$. Human oral epithelial cells (EC) (TERT-2 OKF-6, BWH Cell Culture and Microscopy Core, Boston, MA, USA) were cultured in Keratinocyte-SFM medium (Life Technologies, Saint-Aubin, France). To reduce the risk of contamination, 100 units/mL of penicillin and 100 µg/mL of streptomycin (PromoCell, Heildelberg, Germany) were added to cell media. Cells were grown at 37 °C in a humidified atmosphere with 5% CO_2 and media were changed each 3 days. The number of viable EC was determined using a trypan blue dye exclusion test. Ten µL (2×10^5 cells/ml) of each cell sample was acquired and mixed with 10 µL of 0.5% trypan blue solution (Bio-Rad Laboratories, Inc. Hercules, CA, USA). Ten µL of the mix were acquired and placed on a dual chamber cell counting slide (Bio-Rad Laboratories, Inc. Hercules, CA, USA). The cell counting slide was then loaded in a TC20 automated cell counter (Bio-Rad Laboratories, Inc. Hercules, CA, USA) to determine the proportion of the cells which excluded the dye. Results were presented as the percent ratio of viable cells to the

total number of cells in the sample as described previously [1]. M101 concentrations up to 1g/L were non-cytotoxic for EC.

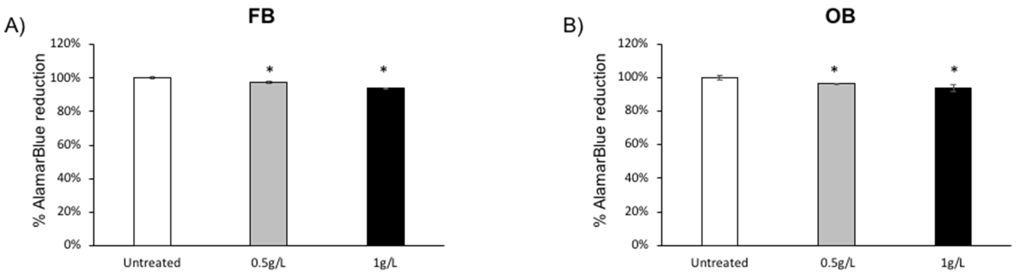

Supplementary figure 2: Metabolic activity of A) human oral fibroblasts (FB) and B) human osteoblasts (OB) with exposure to M101 (0.5, and 1g/L) at 6 hours. % Alamarblue reduction ≤ 20% is not considered cytotoxic. Data are expressed as the mean ± SD. * Difference versus untreated FB and OB, $p<0.05$. Primary gingival fibroblasts (FB) were purchased from American Type Culture Collection (ATCC) (Manassas, VA, US) and cultured in RPMI 1640 cell media (Thermofisher Scientific, Illkirch, France). Human osteoblasts (OB) were obtained from Promocell (Heidelberg, Germany) and cultured in human osteoblasts growth medium (Sigma, St. Quentin Fallavier, France). To reduce the risk of contamination, 100 units/mL of penicillin and 100 μg/mL of streptomycin (PromoCell, Heildelberg, Germany) were added to all cell media. Cells were grown at 37 °C in a humidified atmosphere with 5% CO_2 and media were changed each 3 days. To assess the effect of different concentrations of M101 on FB and OB metabolic activity, an AlamarBlue assay (Life Technologies, Saint-Aubin, France) was performed. Twenty-four hours before the experiment, $2x10^5$ FB and OB were seeded in each well of a 24-well plate. On the day of the experiment, cells were washed twice with PBS. 20 μL of M101 stock solution (50g/L) was added to each well of a 24-well plate with 1mL volume per well (final concentration of M101 as 1g/L). Likewise, volumes of M101 stock solution were adapted to reach final concentrations of 0.5g/L and 1g/L in each well. After treating each cell type (FB, OB) with a range of M101 concentrations (0.5g/L, 1g/L) for 6 hours, 200 μL of

incubation media from all cell types were transferred separately to a 96-well plate and absorbance was measured at OD_{570} and OD_{595} nm by a spectrophotometer (Multiskan, ThermoScientific) in order to determine the percentage of AlamarBlue reduction. M101 concentrations up to 1g/L were non-cytotoxic for both FB and OB.

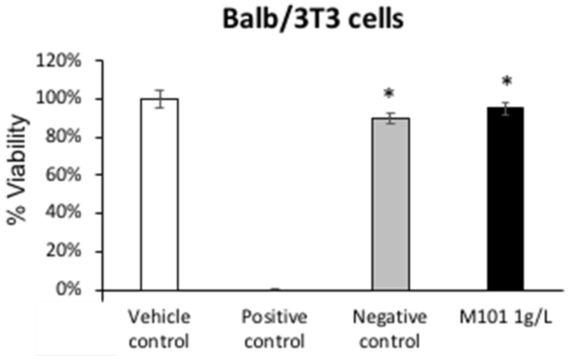

Supplementary figure 3: Percentage viability of BALB/3T3 cells with exposure to M101 (1g/L). % viability reduction ≤ 20% is not considered cytotoxic. Data are expressed as the mean ± SD. * Difference versus untreated BALB/3T3 cells, p<0.05. A toxicological analysis of M101 was carried out on mammalian fibroblasts BALB/3T3 clone A31 (ATCC® CCL-163™). The cells were treated with M101 (1g/L) and incubated at 37 °C in a humidified atmosphere with 5% CO_2 for 24 hours. A quantitative evaluation was performed using the Neutral Red Uptake method (NRU). The cells were treated for 3 hours with the medium containing the cell viability dye and then with a Desorb solution that allows to obtain a cell lysate. The optical density was then calculated after a 540nm spectrophotometric reading. The cells treated with M101 showed a viability reduction of 5%. On the basis of the results, interpreted according to ISO 10993-5:2009 (Biological evaluation of medical devicesPart 5: Tests for in vitro cytotoxicity), M101 must be considered non-cytotoxic.

Proteomic array

To evaluate the impact of M101 treatment on the protein expression of cytokines in EC infected by *P.gingivalis* (MOI=100), a Human Inflammation Antibody Array - Membrane (Abcam ab134003, Paris, France) was used according to manufacturer's instructions. Following targets were analyzed: Eotaxin, Eotaxin-2, GCSF, GM-CSF, ICAM-1, IFN-gamma, I-309, IL-1alpha, IL-1beta, IL-2, IL-3, IL-4, IL-6, IL-6sR, IL-7, IL-8, IL-10, IL-11, IL-12p40, IL-12p70, IL-13, IL-15, IL-16, IL-17, IP-10, MCP-1, MCP-2, M-CSF, MIG, MIP-1alpha, MIP-1beta, MIP-1delta, RANTES, TGF-beta1, TNF-alpha, TNF-beta, sTNF RI, sTNF-RII, PDGF-BB, TIMP-2. Prior to the experiments, $2x10^5$ EC previously infected with *P.gingivalis* MOI=100 were treated with M101 at 1g/L for 6 hours. Then, supernatants were collected and subjected to proteomics analysis. A chemiluminescent blot imaging system (iBright CL1500 imaging system, ThermoFisher Scientific) was used to measure the signal. Signal acquisitions were performed at multiple exposure times between 5 to 50 seconds to obtain optimal signal and interpretation was performed with a densitometry quantification software (LI-COR Image Studio™ Lite).

Reference

1. Castellanos. S. I., Jeremic. A., Cohen. J., and Zderic, V. Ultrasound Stimulation of Insulin Release from Pancreatic Beta Cells as a Potential Novel Treatment for Type 2 Diabetes. *Ultrasound Med Biol* **43**, 1210–1222. (2017).
